# Relationship between Copper, Zinc, and Copper-to-Zinc Ratio in Hair and Severity of Coronary Artery Disease according to the SYNTAX Score

**DOI:** 10.3390/biology12111407

**Published:** 2023-11-07

**Authors:** Ewelina A. Dziedzic, Jakub S. Gąsior, Agnieszka Tuzimek, Ewa Czestkowska, Joanna Beck, Beata Jaczewska, Elżbieta Zgnilec, Andrzej Osiecki, Mirosław Kwaśny, Marek J. Dąbrowski, Wacław Kochman

**Affiliations:** 1 Cardiovascular Clinic, Centre of Postgraduate Medical Education, 01-813 Warsaw, Poland; 2 Department of Pediatric Cardiology and General Pediatrics, Medical University of Warsaw, 02-091 Warsaw, Poland; 3 Nencki Institute of Experimental Biology, Polish Academy of Sciences, 00-901 Warsaw, Poland; 4 Medical Faculty, Lazarski University, 02-662 Warsaw, Poland; 5 Military Institute of Aviation Medicine, 01-755 Warsaw, Poland; 6 Institute of Optoelectronics, Military University of Technology, 00-908 Warsaw, Poland; 7 Department of Cardiology, Bielanski Hospital, 01-809 Warsaw, Poland

**Keywords:** zinc, copper, copper-to-zinc ratio, atherosclerosis, coronary artery disease, ischemic heart disease, SYNTAX score

## Abstract

**Simple Summary:**

Coronary artery disease is a major cause of death worldwide, so it is important to find new changeable factors to prevent it. Some recent studies suggest that not having enough zinc and copper in the body might make plaque build up in heart arteries, which effects in coronary artery disease. In this study, we wanted to discover whether the amount of copper and zinc in hair could tell us something about plaque buildup. We looked at 130 patients and used a scoring system called the SYNTAX score to see how severe the plaque buildup in their heart arteries was. We also checked the copper and zinc levels in their hair and the ratio between these elements. The results showed that lower copper levels in hair and a lower copper-to-zinc ratio were linked to worse plaque buildup in heart arteries. However, we did not find a connection between zinc levels in hair and the severity of plaque buildup. Using hair samples might help us learn more about how copper and zinc affect coronary artery disease, but there is a need for more studies on this topic.

**Abstract:**

Coronary artery disease (CAD) continues to be a foremost contributor to global mortality, and the quest for modifiable risk factors could improve prophylactic strategies. Recent studies suggest a significant role of zinc (Zn) and copper (Cu) deficiency in atheromatous plaque formation. Furthermore, hair was previously described as a valuable source of information on elemental burden during the 6–8 week period before sampling. The aim of this study was to investigate the possibility of correlation between the extent of CAD evaluated with the SYNergy Between PCI With TAXUS and the Cardiac Surgery (SYNTAX) score with Cu and Zn content in hair samples, as well as with the Cu/Zn ratio in a cohort of 130 patients. Our findings describe a statistically significant inverse correlation between Cu content and the Cu/Zn ratio in hair samples and the extent of CAD. In contrast, no significant correlation was found between Zn content and the extent of CAD. Considering the scarcity of existing data on the subject, the analysis of hair samples could yield a novel insight into elemental deficiencies and their potential influence on CAD extent.

## 1. Introduction

Coronary artery disease (CAD) continues to be a persistent, widespread mortality cause despite collective efforts to promote well-being and, thus, mitigate cardiovascular risk factors. According to the World Health Organization (WHO), recent years brought an increase in CAD mortality, with the annual toll reaching 9 million [1]. The underlying pathomechanism of CAD, atherosclerosis, is a chronic process involving inflammatory, necrotic, and smooth muscle cells, and lipid accumulation and transformation beneath the single-cell layer of endothelium. The mechanism of atherosclerotic plaque onset remains not fully understood. The scope of potentially modifiable factors remains extensive, rendering their discovery universally beneficial [2]. Recent investigations proposed that zinc (Zn) [3] and copper (Cu) [4] deficiency, as well as their respective ratio [5], might play a pivotal role in the pathogenesis of atherosclerosis.

Zn is a crucial micronutrient for the maintenance of healthy physiological functioning [6]. Intracellular Zn resides in organelles and vesicles, resulting in a low serum concentration in the range between 10 µmol/L and pmol/L [7]. Zn interacts with cells essential to the pathogenesis of atherosclerosis, including endothelium, smooth muscle cells, and immune cells [3]. However, the role of Zn in the pathogenesis of CAD remains to be fully established.

Cu is a trace element with a significant impact on human physiological processes, including the regulation of iron mobilization, antioxidant protection, and blood clotting [8]. Dysregulated Cu homeostasis was implicated to factor in cardiovascular disease (CVD) onset through diverse mechanisms. The primary source of Cu is oral ingestion with food, with the absorption levels considerably depending on the meal composition [9]. Considering that Cu and Zn absorption is dependent on many factors, including but not limited to sex, age, medication, and supplement intake [10], serum concentration could prove to be a less precise indicator of the elemental status.

Hair microelement analysis offers a unique vantage point of elemental burden evaluation between the last four and eight weeks before sampling [11]. In contrast to blood and urine analyses, hair testing evaluates element levels without the disadvantage of short-term variability [12]. Furthermore, this method is also a painless and noninvasive procedure [13].

Recent investigations aiming to determine the impact of Zn and Cu levels on CVD have yielded disparate outcomes. The majority of the analyses have described a negative influence of Zn deficiency on the incidence of CVD [14,15,16,17]. Nonetheless, a subset of studies found a positive correlation [18] or, in some instances, a lack of the correlation [19,20]. Similar inconsistent results have been described regarding the correlation between Cu and the Cu to Zn (Cu/Zn) ratio with CVD [18,21,22,23].

Our previous analyses failed to reveal differences in the concentration of Zn and Cu and the Cu/Zn ratio when comparing patients with acute coronary syndrome (ACS) to those diagnosed with CAD [19,21]. These parameters were similar across patients displaying differing degrees of atherosclerosis. It is worth pointing out that the severity of CAD was previously described using the simplistic Coronary Artery Surgery Study Scale (CASSS), which constituted a notable limitation for the previously presented results. Hence, the objective of this investigation was to assess the correlation between Zn and Cu concentration, as well as with the Cu/Zn ratio, in hair samples obtained from patients with CAD, utilizing the more comprehensive SYNergy Between PCI With TAXUS and Cardiac Surgery (SYNTAX) scale for evaluating CAD severity. According to the European Society of Cardiology (ESC), this scale is a fundamental tool for the comprehensive assessment of CAD and the long-term prognosis. Additionally, it also plays a crucial role in the selection of the revascularization method in patients afflicted with complex CAD [24].

## 2. Materials and Methods

### 2.1. Population

This analysis was based on a cohort of patients who were admitted to the Department of Cardiology of Bielanski Hospital (Warsaw, Poland) during the period of 2013 to 2017 for the evaluation of CAD using coronary angiography. All patients were residents of Warsaw, Poland, with no history of occupational exposure to chemical elements. They were subjected to coronary angiography due to suspected ACS. Each patient agreed in a written consent form to use their data in research. Individuals with significantly elevated inflammatory markers, active neoplastic diseases, paraneoplastic syndromes, viral or bacterial infection, chronic kidney disease (stages III–V), or who were dying or permanently waving hair in a 3 cm segment counting from the scalp, using any hair product with an increased content of Cu or Zn, and ingesting medications or dietary supplements containing those elements were excluded from the study. This cohort was also previously described in other studies [19,21]. The study was approved by the Medical University of Warsaw bioethics committee and was carried out in accordance with the Declaration of Helsinki.

### 2.2. Laboratory and Clinical Data

Clinical, laboratory, and anthropometric data from patient files were used to determine diagnoses of obesity, overweight, hyperlipidemia, type 2 diabetes mellitus (t2DM), and hypertension (HTN). BMI was calculated as the ratio of weight (kg) to the square of height (m^2^) to diagnose obesity or overweight according to the European Guidelines for Obesity Management in Adults [25]. Blood samples were taken on the day of admission from the cephalic vein and used to perform laboratory tests with standard hospital procedures, including serum levels of total cholesterol (TC), high-density lipoprotein cholesterol (HDL), triglycerides (TG), and glucose. Low-density lipoprotein cholesterol (LDL) was calculated with the Friedewald formula. The 2019 ESC Guidelines for the management of dyslipidemias were used to assess if the patient did not meet the treatment goals for their risk level and diagnose hyperlipidemia [26]. T2DM was diagnosed if two measurements of the fasting blood glucose level exceeded 7.0 mmol/L (126 mg/dL), or blood glucose at 120 min during an oral glucose tolerance test exceeded 11.1 mmol/L (200 mg/dL), or random blood glucose levels exceeded 11.1 mmol/L (200 mg/dL), accompanied by signs and symptoms of hyperglycemia [27]. The 2021 European Society of Hypertension Practice Guidelines criteria (blood pressure exceeding 140/90 mmHg during two in-office measurements) were used to diagnose HTN [28].

### 2.3. Sample Collection and Analysis

Hair samples, weighing between 200 and 300 mg, were obtained from a few separate scalp sites at the back of the head, close to the skin. Subsequently, they were washed for 5 min in an ultrasonic bath with water with a non-ionic detergent (Triton X-100, Sigma Aldrich, Poznań, Poland) in a 1:100 dilution, rinsed with high purity water, acetone, and water, and then dried. Solid samples, 150 mg each, were placed in a closed polypropylene vial (8 mL) and dissolved in a mixture of 4 mL of 65% nitric acid (Merck, Darmstadt, Germany) and 1 mL of 30% hydrogen peroxide (Merck, Darmstadt, Germany), and incubated at 80 °C for 30 min in a microwave station. The samples were then cooled to room temperature and diluted to a final volume of 10 mL with Milli-Q water and analyzed with a previously validated method using an ICP-OES spectrometer (iCAP7400, Thermo Scientific, Waltham, MA, USA) [29]. The concentrations of Cu and Zn in the solutions were calculated on the basis of the results obtained for certified standards: CGZN1 and CGCU1 (Inorganic Ventures, Christiansburg, VA, USA) for Zn and Cu, respectively, resulting finally in a total element content in the samples.

### 2.4. Coronary Angiography

Coronary angiography is an invasive diagnostic and potentially therapeutic procedure using X-rays and iodine contrast to visualize stenosis in the arteries. The data collected during coronary angiographies with access through the radial or femoral arteries were used to assess the extent and complexity of CAD with the SYNTAX score. The SYNTAX score is derived through the application of an algorithm that incorporates the information obtained from coronary angiogram images. This algorithm takes into account the parameters based on the quantity of arterial lesions, their spatial distribution within the coronary vasculature, and their respective effects on hemodynamic blood flow. The SYNTAX score was found to be an independent predictor of long-term major adverse cardiac and cerebrovascular events, and could be considered as an effect modifier when choosing treatment [24]. Subsequent treatment was performed if necessary, as instructed by the ESC guidelines on the management of stable CAD and the guidelines on myocardial revascularization applicable at that time [30,31].

### 2.5. Statistical Analysis

The data distribution was determined using a Shapiro–Wilk test. For data not normally distributed, the median with the interquartile range (IQR 25–75) was presented. Multivariate ordinal logistic regression was performed to investigate the factors influencing the Cu, Zn, and Cu/Zn level. The relationship between the selected variables was analyzed with a Spearman correlation coefficient (R). Statistical significance was recognized if a two-sided *p*-value < 0.05. Analysis was performed using Statistica 13.3 software (TIBCO Software Inc., Palo Alto, CA, USA).

## 3. Results

### 3.1. Study Population

The results of 130 (N = 36, 27.7% females; N = 94, 72.3% males) participants with a median age of 65 years (IQR: 60–75) were presented in the study. The median BMI value was 28 kg/m^2^ (IQR: 25–31). A total of 38 (29.2%) participants had a normal body weight, 52 (40.0%) were overweight, and 40 (30.8%) patients were classified as obese.

Active smoking during the study was declared by 38 (29.2%) patients, 17 (13.1%) patients had smoked in the past, and 75 (57.7%) patients were no-smokers.

Hypertension was present in 112 (86.2%) patients and no hypertension was present in only 18 (13.8%) patients.

A history of type 2 diabetes mellitus (t2DM) or diagnosis during the current hospitalization was found in 41 (31.5%) patients, pre-diabetes was found in 7 (5.4%) patients, and no t2DM diagnosis was found in 82 (63.1%) patients.

On the basis of the lipid profile (total cholesterol—TC: median and IQR: 165 mg/dL, 137–197; LDL: median and IQR: 89 mg/dL, 69–126; HDL: median and IQR: 46 mg/dL, 40–54; triglycerides—TG: median and IQR: 110 mg/dL, 88–152), hyperlipidemia was assessed in 120 patients and diagnosed in 53 (40.8%). A history of myocardial infarction (MI) was noticed in 38 (29.2%) patients. Acute coronary syndrome (ACS) as the cause of hospitalization was diagnosed in 65 (50.0%) patients (STEMI N = 31, 23.9%; NSTEMI N = 19, 14.6%; UA N = 15, 11.5%), whereas stable CAD was diagnosed in 65 (50.0%) patients. The median SYNTAX score was 14 points (IQR: 5–26).

The median Cu, Zn, and Cu/Zn concentrations were as follows: 9.0 parts per million (ppm) (IQR: 7.3–11.0), 168 ppm (IQR: 139–191), and 0.05 (IQR: 0.04–0.07).

Females were statistically older and presented higher values of TC and HDL than males (*p* < 0.05 for all). Statistically, more males were active smokers (*p* < 0.05). There were no significant differences between males and females in other analyzed parameters.

### 3.2. Determinants of Cu, Zn, and Cu/Zn

Cu, Zn, and Cu/Zn were divided into four quartiles and used in multivariate ordinal logistic regression analyses as four-level dependent variables (Table 1).

The results of the multivariate ordinal logistic regression analyses of factors associated with the Cu, Zn, and Cu/Zn level are presented in Table 2, Table 3 and Table 4. The SYNTAX score was negatively associated with the Cu and Cu/Zn level, whereas diabetes was associated with the Zn level. The Zn level was lower in patients with diabetes than those without or with a pre-diabetes status.

### 3.3. Association between Cu, Zn, Cu/Zn-Ratio, and Severity of CAD

**Figure 1:**
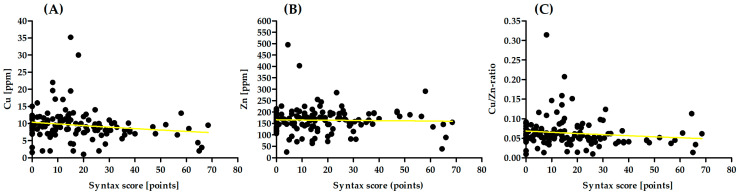
Association between (**A**) Cu, (**B**) Zn, (**C**) Cu/Zn-Ratio, and Severity of CAD.

There was no significant correlation between Zn and the SYNTAX score (R = 0.05, *p* = 0.547, Figure 1A). There was a significant correlation between Cu and the SYNTAX score (R = −0.19, *p* = 0.032, Figure 1B), and also between the Cu/Zn-Ratio and severity of CAD assessed using SYNTAX (R = −0.20, *p* = 0.025, Figure 1C).

## 4. Discussion

The results of this study did not find a correlation between the Zn content in hair samples and the complexity of CAD assessed with the SYNTAX score. However, a statistically significant inverse correlation was described between the SYNTAX score and the Cu content in hair, as well as the Cu/Zn ratio. Notably, the previous observations of a lack of correlation between Zn content and the severity of CAD were documented [19]. The principal limitation of the mentioned research stemmed from the utilization of the four-grade CASS scale, which, while being characterized by its simplicity and qualitative utility, did not fully reflect the multifaceted nature of CAD. The CASSS offered a rudimentary classification between one-, two-, and three-vessel disease. The present analysis utilized a more refined and sophisticated SYNTAX scale, which has well-established scientific and clinical value. Notwithstanding this advancement, the application of the SYNTAX scale has supported our previous conclusions about the absence of a significant correlation between Zn content in hair and the severity of CAD. Furthermore, the findings demonstrated that lower values of the Cu/Zn ratio were linked to a higher SYNTAX score.

The subject of Cu and Zn levels and the complexity of CAD has received limited attention so far, with the vast majority of studies relying on serum or urine analyses. Furthermore, the results across the studies have displayed notable inconsistencies, rendering them ultimately inconclusive in determining if the Zn and Cu content is correlated with the severity of CAD [32,33,34,35]. A comparative analysis of the Zn and Cu levels between patients with and without CAD (67 vs. 26 patients) revealed a significant correlation with the diagnosis of CAD, but no correlation was observed with the extent of the disease [32]. Similar results were described by Lim et al., as they have not found a correlation between serum Cu levels and the severity of CAD [33]. Despite the consistent conclusions of the aforementioned studies with our results on Zn content [32], our data contradicts the absence of correlation between Cu levels and CAD complexity, as reported in the aforementioned reports [32,33]. This discrepancy could be possibly attributed to the variations in the scoring systems used to assess the severity of CAD (Gensini [32,36], number of affected arteries [33]), as well as the diversity of the materials and methods used to measure the transient concentration of microelements. On the other hand, Giannoglou et al. also did not identify a correlation between Zn serum concentration and CAD severity, but did report a positive correlation between Zn excretion through urine and CAD extent. It is important to note that the majority of the patients were treated with thiazide diuretics, which significantly affect the urinary excretion of Zn [34]. Mielcarz et al. described an interesting perspective on Zn and Cu content as well as the Cu/Zn ratio by measuring the microelement content not only in serum, but also in leukocyte DNA and proteins. Although they did not observe a significant correlation between Zn and Cu levels, as well as between the Cu/Zn ratio in serum and CAD, they described a correlation between the Cu levels in leukocytes and the extent of CAD [37]. This analysis highlights the dependence of the results obtained on the type of biological sample used to measure the levels of microelements. An analysis of the serum Cu content in 337 patients with CAD by Bagheri et al. revealed a positive correlation between this microelement and the SYNTAX score, contradicting the results presented in this study [35]. Despite the same scale being used for the assessment of CAD severity, the ethnic differences between cohorts and different sample materials challenge a straightforward comparison between the results. In agreement with our findings, Mahalle et al. described a relation between lower Zn and Cu food intake and the severity of CAD [38]. However, their approach was based on a dietary interview from two consecutive days as an assessment of microelement content, without verification through laboratory testing. Thus, comparing these results with our data seems difficult, given that hair samples are considered to reflect the exposure to microelements over the previous two months [11].

There is a limited number of studies that assess the relationships of the Zn and Cu content in hair samples and CVD. These studies typically aimed to correlate these microelements with ACS, rather than with the complexity and severity of CAD. Due to the inconsistent results of these analyses, the question of the correlation between the Zn and Cu levels in hair and ACS remains unresolved [39,40,41,42,43,44,45,46].

The existing literature exploring the relationship between traditional CAD risk factors and Zn, Cu, and their proportions is limited [47]. Our findings indicate a notable decrease in zinc content in the hair samples from individuals with t2DM, aligning with experimental studies that emphasize the pivotal role of zinc in glucose metabolism. Zinc is thought to be involved in insulin release mechanisms within the β-cells of the islets of Langerhans [48], gluconeogenesis [49,50,51], as well as in the regulation of tissue sensitivity to insulin [52]. Furthermore, its supplementation was suggested to have potential benefits for patients with t2DM [50,53]. In our study cohort, the hair Cu content and the Cu/Zn ratio were not significantly different among patients with and without t2DM. The findings from other studies are inconclusive; some are consistent with our results [54], while others suggest a negative correlation between hair Cu content and glycated hemoglobin levels [55]. Additionally, Cu was reported to exhibit content-dependent effects, with deficiency or excess having potential detrimental impacts on human health [56,57]. These observations emphasize the need for further well-designed research to elucidate potential indications for supplementation.

Within our examined group, we did not observe any correlation between HTN and the levels of Zn, Cu, or the Cu/Zn ratio in hair samples. This may be attributed to the demographics of our analyzed group, given that a significant majority of our patients had well-controlled HTN. A recent comparative analysis involving nearly 400 hair samples from individuals diagnosed with HTN and healthy individuals revealed lower Cu and Zn content in the hair of those with HTN [58]. However, these results were not corroborated by Vivoli et al., who reported no differences in hair Cu and Zn content between corresponding groups [59]. It is essential to recognize that these previously mentioned analyses cannot be directly compared to our findings, as our cohort consists of patients with CAD. Considering the intricate involvement of extracellular and intracellular Zn in various mechanisms [60], its J-shaped correlation between dietary Zn intake and HTN diagnosis [61], as well as the multifaceted effects of Cu on the cardiovascular system, it is fundamental to conduct comprehensive research to thoroughly investigate the roles of both elements in the context of HTN and blood pressure regulation.

Our data indicate that there is no evident correlation between hair Cu or Zn content and hyperlipidemia. This finding appears to contradict a recent comprehensive meta-analysis that spanned studies conducted between 1990 and 2022 [47], which focused on the relationship between serum trace elements and lipid metabolism. The aforementioned study reported a lack of correlation between serum Zn levels and dyslipidemia, but it did identify a significant correlation with other elements, including Cu. It is worth noting that this inconsistency may be attributed to the analysis of different sample types, as the studies examined in the meta-analysis employed serum samples, making direct result comparisons with our results challenging. Furthermore, most data in the aforementioned meta-analysis were derived from comparative studies involving healthy individuals and patients, whereas our research centered on patients who were already undergoing treatment with hypolipemic medications.

We did not detect a significant correlation between body weight or smoking habits and hair Cu or Zn levels. Given the incongruous findings from the available studies conducted on healthy individuals, drawing definitive conclusions on this subject remains a matter of ongoing research [45,46,62,63,64].

In summary, the correlation between traditional CAD risk factors and hair Zn and Cu content and the Zn/Cu ratio necessitates further well-designed research due to the inconsistencies observed in the methods and results of existing studies.

This study has its limitations, which should be acknowledged when interpreting the findings, and may warrant further investigation and considerations in future research endeavors. Its cross-sectional and observational character precludes the ability to determine causal relationships among the analyzed variables. The cohort exhibits limited demographic diversity and this constraint is associated with the cost of sample analysis. The potential influence of medications, including angiotensin-converting enzyme inhibitors, angiotensin receptor blockers, diuretics, and β-adrenolytics, was not accounted for in the analysis. Furthermore, the assessment of microelement content was evaluated exclusively in hair samples, without comparative evaluation across different types of samples (serum, urine, and leukocytes) and measuring methods.

The concept of hair analysis leverages the advantages associated with a relatively consistent rate of hair growth, approximately 1 cm per month. Hair serves as a repository for xenobiotics, primarily derived from the bloodstream [65]. Consequently, the analysis of xenobiotics in hair offers additional opportunities for assessing mineral nutrition and monitoring exposure to toxins or environmental contaminants [66,67]. Given the pivotal role of Cu and Zn in various essential biological processes, numerous studies have sought to explore the incorporation of microelements into hair, any potential alterations in hair structure or composition, and their associations with biogenic factors [65]. Considering the prolonged inflammation of the vascular wall, a hallmark of chronic coronary syndrome, the use of hair as a biological material enables the assessment of the long-term exposure to specific substances, as opposed to the transient concentrations in serum or urine.

The reference values for the Zn or Cu content in hair and Cu/Zn ratio have not been established yet. Furthermore, the SYNTAX score was postulated to have an inherent limitation related to inter-individual variability in its calculation [68].

In summary, this study identified a statistically significant inverse correlation between the complexity of CAD, as assessed with the SYNTAX score, and both Cu content and the Cu/Zn ratio in hair samples. Conversely, the Zn levels did not correlate with the SYNTAX score. The observed variance of microelement levels across different sample types (serum, urine, hair, leukocytes) in various studies suggests that these nutrients are involved in complex processes, emphasizing the need for further well-designed research to comprehensively explain their role in the pathogenesis of atherosclerosis. Considering the ease and noninvasiveness of obtaining hair samples, their analysis could potentially become a routine practice in clinical settings, providing prior extensive research.

## 5. Conclusions

In this study, an inverse correlation was observed between the extent of CAD assessed with the SYNTAX scale and Cu content, as well as the Cu/Zn ratio in hair samples. Conversely, the Zn content in the hair samples did not exhibit a significant correlation with the SYNTAX score in the cohort of patients with confirmed CAD, as determined through coronary angiography.

## Figures and Tables

**Table 1:** Quartiles for Cu, Zn, and Cu/Zn.

	Q1 < 25%	Q2 25–50%	Q3 50–75%	Q4 > 75%
Cu	6.6 (1.0–7.3)	8.4 (7.4–9.0)	10.0 (9.1–11.0)	12.7 (11.2–35.2)
Zn	113 (25–139)	152 (140–167)	176 (168–191)	206 (192–495)
Cu/Zn	0.04 (0.01–0.04)	0.05 (0.05–0.05)	0.06 (0.05–0.07)	0.09 (0.07–0.31)

**Table 2:** Multivariate ordinal logistic regression analysis of factors associated with Cu level.

Variables	Effect for	β	95% CI	Wald Stat.	*p*-Value
Age	-	0.013	−0.011–0.036	1.104	0.294
Sex	Males	−0.004	−0.487–0.480	<0.001	0.989
BMI	-	0.005	−0.037–0.046	0.05	0.827
Smoking	Smokers	−0.103	−0.579–0.373	0.179	0.672
Hypertension	Yes	0.042	−0.554–0.639	0.019	0.889
Diabetes	Yes	−0.248	−0.701–0.205	1.150	0.284
	Pre-diabetes	1.123	0.030–2.217	4.053	0.044
Hyperlipidemia	Yes	0.106	−0.299–0.511	0.262	0.609
Previous MI	Yes	0.158	−0.294–0.609	0.469	0.493
Diagnosis	ACS	0.151	−0.262–0.563	0.513	0.474
SYNTAX score	-	−0.019	−0.033–−0.006	8.29	0.004

**Table 3:** Multivariate ordinal logistic regression analysis of factors associated with Zn level.

Variables	Effect for	β	95% CI	Wald Stat.	*p*-Value
Age	-	−0.006	−0.029–0.018	0.241	0.623
Sex	Males	0.291	−0.198–0.780	1.360	0.244
BMI	-	−0.027	−0.070–0.015	1.584	0.208
Smoking	Smokers	−0.410	−0.894–0.073	2.764	0.096
Hypertension	Yes	−0.127	−0.726–0.472	0.173	0.678
Diabetes	Yes	−0.689	−1.153–−0.226	8.484	0.004
	Pre-diabetes	−0.156	−1.195–0.883	0.086	0.769
Hyperlipidemia	Yes	0.004	−0.405–0.414	<0.001	0.983
Previous MI	Yes	0.075	−0.383–0.533	0.103	0.748
Diagnosis	ACS	−0.004	−0.421–0.414	<0.001	0.986
SYNTAX score	-	0.003	−0.010–0.016	0.165	0.685

**Table 4:** Multivariate ordinal logistic regression analysis of factors associated with Cu/Zn level.

Variables	Effect for	β	95% CI	Wald Stat.	*p*-Value
Age	-	0.006	−0.018–0.029	0.229	0.632
Sex	Males	−0.082	−0.566–0.401	0.112	0.738
BMI	-	0.024	−0.018–0.066	1.23	0.267
Smoking	Smokers	0.065	−0.409–0.539	0.073	0.788
Hypertension	Yes	−0.028	−0.621–0.565	0.008	0.927
Diabetes	Yes	0.112	−0.341–0.565	0.236	0.627
	Pre-diabetes	0.409	−0.631–1.449	0.593	0.441
Hyperlipidemia	Yes	0.176	−0.229–0.580	0.724	0.395
Previous MI	Yes	0.309	−0.144–0.762	1.789	0.181
Diagnosis	ACS	0.256	−0.159–0.670	1.462	0.227
SYNTAX score	-	−0.017	−0.030–−0.004	6.221	0.013

## Data Availability

Data can be provided by the corresponding author upon reasonable request.

## References

[1] World Health Organisation Global Health Estimates: Life Expectancy and Leading Causes of Death and Disability. www.who.int/data/gho/data/themes/mortality-and-global-health-estimates.

[2] Björkegren J.L.M., Lusis A.J. (2022). Atherosclerosis: Recent developments. Cell.

[3] Shen T., Zhao Q., Luo Y., Wang T. (2022). Investigating the Role of Zinc in Atherosclerosis: A Review. Biomolecules.

[4] Chen X., Cai Q., Liang R., Zhang D., Liu X., Zhang M., Xiong Y., Xu M., Liu Q., Li P. (2023). Copper homeostasis and copper-induced cell death in the pathogenesis of cardiovascular disease and therapeutic strategies. Cell Death Dis..

[5] Malavolta M., Giacconi R., Piacenza F., Santarelli L., Cipriano C., Costarelli L., Tesei S., Pierpaoli S., Basso A., Galeazzi R. (2010). Plasma copper/zinc ratio: An inflammatory/nutritional biomarker as predictor of all-cause mortality in elderly population. Biogerontology.

[6] Stefanidou M., Maravelias C., Dona A., Spiliopoulou C. (2006). Zinc: A multipurpose trace element. Arch. Toxicol..

[7] Outten C.E., O’Halloran T.V. (2001). Femtomolar sensitivity of metalloregulatory proteins controlling zinc homeostasis. Science.

[8] Liu Y., Miao J. (2022). An Emerging Role of Defective Copper Metabolism in Heart Disease. Nutrients.

[9] Linder M.C., Hazegh-Azam M. (1996). Copper biochemistry and molecular biology. Am. J. Clin. Nutr..

[10] Bost M., Houdart S., Oberli M., Kalonji E., Huneau J.-F., Margaritis I. (2016). Dietary copper and human health: Current evidence and unresolved issues. J. Trace Elem. Med. Biol..

[11] Nakaona L., Maseka K.K., Hamilton E.M., Watts M.J. (2020). Using human hair and nails as biomarkers to assess exposure of potentially harmful elements to populations living near mine waste dumps. Environ. Geochem. Health.

[12] Chojnacka K., Górecka H., Chojnacki A., Górecki H. (2005). Inter-element interactions in human hair. Environ. Toxicol. Pharmacol..

[13] Çelik B., Nalçacıoğlu H., Karakükçü Ç., Aslaner H., Şahiner Ü.M. (2020). Assessment of Hair Zinc in the School Children in Kayseri, Turkey. Biol. Trace Elem. Res..

[14] Kazemi-Bajestani S.M.R., Ghayour-Mobarhan M., Ebrahimi M., Moohebati M., Esmaeili H.A., Parizadeh M.R., Aghacizadeh R., Ferns G.A.A. (2007). Serum copper and zinc concentrations are lower in Iranian patients with angiographically defined coronary artery disease than in subjects with a normal angiogram. J. Trace Elem. Med. Biol..

[15] Choi S., Liu X., Pan Z. (2018). Zinc deficiency and cellular oxidative stress: Prognostic implications in cardiovascular diseases. Acta Pharmacol. Sin..

[16] Liu M., Zhu H., Zhai T., Pan H., Wang L., Yang H., Yan K., Zeng Y., Gong F. (2019). Serum Zinc-α2-Glycoprotein Levels Were Decreased in Patients with Premature Coronary Artery Disease. Front. Endocrinol..

[17] Meng H., Wang Y., Zhou F., Ruan J., Duan M., Wang X., Yu Q., Yang P., Chen W., Meng F. (2021). Reduced Serum Zinc Ion Concentration Is Associated with Coronary Heart Disease. Biol. Trace Elem. Res..

[18] Kärberg K., Forbes A., Lember M. (2022). Raised dietary Zn:Cu ratio increases the risk of atherosclerosis in type 2 diabetes. Clin. Nutr. ESPEN.

[19] Dziedzic E.A., Gąsior J.S., Tuzimek A., Paleczny J., Kwaśny M., Dąbrowski M., Jankowski P. (2022). No Association of Hair Zinc Concentration with Coronary Artery Disease Severity and No Relation with Acute Coronary Syndromes. Biomolecules.

[20] De Paula R.C.S., Aneni E.C., Costa A.P.R., Figueiredo V.N., Moura F.A., Freitas W.M., Quaglia L.A., Santos S.N., Soares A.A., Nadruz W. (2014). Low zinc levels is associated with increased inflammatory activity but not with atherosclerosis, arteriosclerosis or endothelial dysfunction among the very elderly. BBA Clin..

[21] Dziedzic E.A., Tuzimek A., Gąsior J.S., Paleczny J., Junka A., Kwaśny M., Dąbrowski M., Jankowski P. (2022). Investigation on the Association of Copper and Copper-to-Zinc-Ratio in Hair with Acute Coronary Syndrome Occurrence and Its Risk Factors. Nutrients.

[22] Jäger S., Cabral M., Kopp J.F., Hoffmann P., Ng E., Whitfield J.B., Morris A.P., Lind L., Schwerdtle T., Schulze M.B. (2022). Blood copper and risk of cardiometabolic diseases: A Mendelian randomization study. Hum. Mol. Genet..

[23] Urbanowicz T., Hanć A., Olasińska-Wiśniewska A., Rodzki M., Witkowska A., Michalak M., Perek B., Haneya A., Jemielity M. (2022). Serum copper concentration reflect inflammatory activation in the complex coronary artery disease—A pilot study. J. Trace Elem. Med. Biol..

[24] Neumann F.-J., Sousa-Uva M., Ahlsson A., Alfonso F., Banning A.P., Benedetto U., Byrne R.A., Collet J.-P., Falk V., Head S.J. (2019). 2018 ESC/EACTS Guidelines on myocardial revascularization. Eur. Heart J..

[25] Yumuk V., Tsigos C., Fried M., Schindler K., Busetto L., Micic D., Toplak H. (2015). European Guidelines for Obesity Management in Adults. Obes. Facts.

[26] Mach F., Baigent C., Catapano A.L., Koskinas K.C., Casula M., Badimon L., Chapman M.J., de Backer G.G., Delgado V., Ference B.A. (2020). 2019 ESC/EAS Guidelines for the management of dyslipidaemias: Lipid modification to reduce cardiovascular risk. Eur. Heart J..

[27] Araszkiewicz A., Bandurska-Stankiewicz E., Borys S., Budzyński A., Cyganek K., Cypryk K., Czech A., Czupryniak L., Drzewoski J., Dzida G. (2021). 2021 Guidelines on the management of patients with diabetes. A position of Diabetes Poland. Clin. Diabetol..

[28] Stergiou G.S., Palatini P., Parati G., O’Brien E., Januszewicz A., Lurbe E., Persu A., Mancia G., Kreutz R. (2021). 2021 European Society of Hypertension practice guidelines for office and out-of-office blood pressure measurement. J. Hypertens..

[29] Shizhong C., Dengbo L., Zhixiong H., Zhan W. (2005). The use of electrothermal vaporization ICP-OES for the determination of trace elements in human hair using slurry sampling and PTFE as modifier. Int. J. Environ. Anal. Chem..

[30] Montalescot G., Sechtem U., Achenbach S., Andreotti F., Arden C., Budaj A., Bugiardini R., Crea F., Cuisset T., Di Mario C. (2013). 2013 ESC guidelines on the management of stable coronary artery disease: The Task Force on the management of stable coronary artery disease of the European Society of Cardiology. Eur. Heart J..

[31] Windecker S., Kolh P., Alfonso F., Collet J.-P., Cremer J., Falk V., Filippatos G., Hamm C., Head S.J., Jüni P. (2014). 2014 ESC/EACTS Guidelines on myocardial revascularization: The Task Force on Myocardial Revascularization of the European Society of Cardiology (ESC) and the European Association for Cardio-Thoracic Surgery (EACTS)Developed with the special contribution of the European Association of Percutaneous Cardiovascular Interventions (EAPCI). Eur. Heart J..

[32] Islamoglu Y., Evliyaoglu O., Tekbas E., Cil H., Elbey M.A., Atilgan Z., Kaya H., Bilik Z., Akyuz A., Alan S. (2011). The relationship between serum levels of Zn and Cu and severity of coronary atherosclerosis. Biol. Trace Elem. Res..

[33] Lima A., Ferin R., Fontes A., Santos E., Martins D., Baptista J., Pavão M.L. (2021). Circulating antioxidant vitamins and copper in Azorean coronary artery disease patients under preventive medication—A case study. J. Trace Elem. Med. Biol..

[34] Yendt E.R., Cohanim M. (1978). Prevention of calcium stones with thiazides. Kidney Int..

[35] Bagheri B., Akbari N., Tabiban S., Habibi V., Mokhberi V. (2015). Serum level of copper in patients with coronary artery disease. Niger. Med. J..

[36] Gensini G.G. (1983). A more meaningful scoring system for determining the severity of coronary heart disease. Am. J. Cardiol..

[37] Mielcarz G., Howard A.N., Mielcarz B., Williams N.R., Rajput-Williams J., Nigdigar S.V., Stone D.L. (2001). Leucocyte copper, a marker of copper body status is low in coronary artery disease. J. Trace Elem. Med. Biol..

[38] Mahalle N., Garg M.K., Naik S.S., Kulkarni M.V. (2016). Association of dietary factors with severity of coronary artery disease. Clin. Nutr. ESPEN.

[39] Białkowska M., Hoser A., Szostak W.B., Dybczyński R., Sterliński S., Nowicka G., Majchrzak J., Kaczorowski J., Danko B. (1987). Hair zinc and copper concentration in survivors of myocardial infarction. Ann. Nutr. Metab..

[40] Tang Y.-R., Zhang S.-Q., Xiong Y., Zhao Y., Fu H., Zhang H.-P., Xiong K.-M. (2003). Studies of five microelement contents in human serum, hair, and fingernails correlated with aged hypertension and coronary heart disease. Biol. Trace Elem. Res..

[41] Afridi H.I., Kazi T.G., Kazi G.H., Jamali M.K., Shar G.Q. (2006). Essential trace and toxic element distribution in the scalp hair of Pakistani myocardial infarction patients and controls. Biol. Trace Elem. Res..

[42] Tan C., Chen H., Xia C. (2009). The prediction of cardiovascular disease based on trace element contents in hair and a classifier of boosting decision stumps. Biol. Trace Elem. Res..

[43] Chen A., Li G., Liu Y. (2015). Association between copper levels and myocardial infarction: A meta-analysis. Inhal. Toxicol..

[44] Ilyas A., Ahmad H., Shah M.H. (2015). Comparative Study of Elemental Concentrations in the Scalp Hair and Nails of Myocardial Infarction Patients versus Controls from Pakistan. Biol. Trace Elem. Res..

[45] Ilyas A., Shah M.H. (2017). Disparities of Selected Metal Levels in the Blood and Scalp Hair of Ischemia Heart Disease Patients and Healthy Subjects. Biol. Trace Elem. Res..

[46] Ilyas A., Ahmad H., Shah M.H. (2015). Comparative Distribution, Correlation, and Chemometric Analyses of Selected Metals in Scalp Hair of Angina Patients and Healthy Subjects. Biol. Trace Elem. Res..

[47] Li C.-P., Song Y.-X., Lin Z.-J., Ma M.-L., He L.-P. (2023). Essential trace elements in patients with dyslipidemia: A meta-analysis. Curr. Med. Chem..

[48] Ruz M., Carrasco F., Rojas P., Basfi-Fer K., Hernández M.C., Pérez A. (2019). Nutritional Effects of Zinc on Metabolic Syndrome and Type 2 Diabetes: Mechanisms and Main Findings in Human Studies. Biol. Trace Elem. Res..

[49] Safarzad M., Jazi M.S., Kiaei M., Asadi J. (2023). Lower serum zinc level is associated with higher fasting insulin in type 2 diabetes mellitus (T2DM) and relates with disturbed glucagon suppression response in male patients. Prim. Care Diabetes.

[50] Maret W. (2017). Zinc in Pancreatic Islet Biology, Insulin Sensitivity, and Diabetes. Prev. Nutr. Food Sci..

[51] Chabosseau P., Rutter G.A. (2016). Zinc and diabetes. Arch. Biochem. Biophys..

[52] Ferdowsi P.V., Ahuja K.D.K., Beckett J.M., Myers S. (2023). Capsaicin and Zinc Signalling Pathways as Promising Targets for Managing Insulin Resistance and Type 2 Diabetes. Molecules.

[53] Ghaedi K., Ghasempour D., Jowshan M., Zheng M., Ghobadi S., Jafari A. (2023). Effect of zinc supplementation in the management of type 2 diabetes: A grading of recommendations assessment, development, and evaluation-assessed, dose-response meta-analysis of randomized controlled trials. Crit. Rev. Food Sci. Nutr..

[54] Kazi T.G., Afridi H.I., Kazi N., Jamali M.K., Arain M.B., Jalbani N., Kandhro G.A. (2008). Copper, chromium, manganese, iron, nickel, and zinc levels in biological samples of diabetes mellitus patients. Biol. Trace Elem. Res..

[55] Hotta Y., Fujino R., Kimura O., Endo T. (2018). Essential and Non-essential Elements in Scalp Hair of Diabetics: Correlations with Glycated Hemoglobin (HbA1c). Biol. Pharm. Bull..

[56] Eljazzar S., Abu-Hijleh H., Alkhatib D., Sokary S., Ismail S., Al-Jayyousi G.F., Tayyem R. (2023). The Role of Copper Intake in the Development and Management of Type 2 Diabetes: A Systematic Review. Nutrients.

[57] Taneja S.K., Mahajan M., Gupta S., Singh K.P. (1998). Assessment of copper and zinc status in hair and urine of young women descendants of NIDDM parents. Biol. Trace Elem. Res..

[58] Afridi H.I., Kazi T.G., Kazi N., Talpur F.N., Naeemullah, Arain S.S., Brahman K.D., Wadhwa S.K., Shah F. (2013). Distribution of copper, iron, and zinc in biological samples of Pakistani hypertensive patients and referent subjects of different age groups. Clin. Lab..

[59] Vivoli G., Borella P., Bergomi M., Fantuzzi G. (1987). Zinc and copper levels in serum, urine, and hair of humans in relation to blood pressure. Sci. Total Environ..

[60] Ozyildirim S., Baltaci S.B. (2023). Cardiovascular Diseases and Zinc. Biol. Trace Elem. Res..

[61] He P., Li H., Liu M., Zhang Z., Zhang Y., Zhou C., Ye Z., Wu Q., Liang M., Jiang J. (2023). J-shaped association between dietary zinc intake and new-onset hypertension: A nationwide cohort study in China. Front. Med..

[62] Knez M., Pantovic A., Zekovic M., Pavlovic Z., Glibetic M., Zec M. (2019). Is There a Link between Zinc Intake and Status with Plasma Fatty Acid Profile and Desaturase Activities in Dyslipidemic Subjects?. Nutrients.

[63] Knez M., Nikolic M., Zekovic M., Stangoulis J.C., Gurinovic M., Glibetic M. (2017). The influence of food consumption and socio-economic factors on the relationship between zinc and iron intake and status in a healthy population. Public Health Nutr..

[64] Król E., Bogdański P., Suliburska J., Krejpcio Z. (2019). The Relationship between Dietary, Serum and Hair Levels of Minerals (Fe, Zn, Cu) and Glucose Metabolism Indices in Obese Type 2 Diabetic Patients. Biol. Trace Elem. Res..

[65] Kempson I.M., Skinner W.M., Kirkbride K.P. (2007). The occurrence and incorporation of copper and zinc in hair and their potential role as bioindicators: A review. J. Toxicol. Environ. Health B Crit. Rev..

[66] Kales S.N., Goldman R.H. (2002). Mercury exposure: Current concepts, controversies, and a clinic’s experience. J. Occup. Environ. Med..

[67] Samanta G., Chowdhury T.R., Mandal B.K., Biswas B.K., Chowdhury U.K., Basu G.K., Chanda C.R., Lodh D., Chakraborti D. (1999). Flow Injection Hydride Generation Atomic Absorption Spectrometry for Determination of Arsenic in Water and Biological Samples from Arsenic-Affected Districts of West Bengal, India, and Bangladesh. Microchem. J..

[68] Zhang Y.-J., Iqbal J., Campos C.M., Klaveren D.V., Bourantas C.V., Dawkins K.D., Banning A.P., Escaned J., de Vries T., Morel M.-A. (2014). Prognostic value of site SYNTAX score and rationale for combining anatomic and clinical factors in decision making: Insights from the SYNTAX trial. J. Am. Coll. Cardiol..

